# Appropriate Reporting of Exercise Variables in Resistance Training Protocols: Much more than Load and Number of Repetitions

**DOI:** 10.1186/s40798-022-00492-1

**Published:** 2022-07-30

**Authors:** Giuseppe Coratella

**Affiliations:** Department of Biomedical Sciences for Health, Università degli Studi di Milano, Via Giuseppe Colombo 71, 20133 Milan, Italy

**Keywords:** Strength training, Weight training, Muscle-mind connection, Time under tension, Strength, Hypertrophy, Muscle architecture, Range of movement, Eccentric, Inter-set rest

## Abstract

Manipulating resistance training variables is crucial to plan the induced stimuli correctly. When reporting the exercise variables in resistance training protocols, sports scientists and practitioners often refer to the load lifted and the total number of repetitions. The present conceptual review explores all within-exercise variables that may influence the strength and hypertrophic gains, and the changes in muscle architecture. Together with the (1) load and (2) the number of repetitions, (3) performing repetitions to failure or not to failure, (4) the displacement of the load or the range of movement (full or partial), (5) the portion of the partial movement to identify the muscle length at which the exercise is performed, (6) the total time under tension, the duration of each phase and the position of the two isometric phases, (7) whether the concentric, eccentric or concentric-eccentric phase is performed, (8) the use of internal or external focus and (9) the inter-set rest may all have repercussions on the adaptations induced by each resistance exercise. Manipulating one or more variable allows to increase, equalize or decrease the stimuli related to each exercise. Sports scientists and practitioners are invited to list all aforementioned variables for each exercise when reporting resistance training protocols.

## Key Points

- Traditional reporting of resistance exercise is often described as load x number of repetitions.
- Many more within-exercise variables including performing repetitions to failure/not to failure, load displacement/range of movement, time under tension and position of the isometric phases, performing concentric- or eccentric-only or concentric–eccentric phase, attentional focus and inter-set rest may affect the resistance training-induced adaptations in strength or muscle structure.
- Appropriate reporting of the within-exercise variables allows more accurate determination of the technique, the volume and consequently the stimuli provided by each resistance exercise.

## Introduction

Resistance training is widely used to increase muscle strength [1] and induce neural [2] and structural adaptations [3]. In brief, neural adaptations may span from increasing the motor unit recruitment, synchronization and firing rate and decrease the neuromuscular inhibition with the overall effect to increase muscle strength [1], while structural adaptations include increments in muscle size (e.g. muscle volume or cross-sectional area) [4], changes in muscle architecture (e.g. fascicle elongation and/or fascicle angle widening) [5], and reinforcement of the muscle–tendon complex [6].

While the resistance training-induced adaptations are well-acknowledged, these may depend on a myriad of variables that altogether make the training volume. Therefore, understanding what the resistance training volume is made of appears critical to overload, equalize or underload the stimuli along the training period, given that volume seems a key factor in resistance training [7, 8]. Such variables might be classified as between-session, for example the time between two consecutive sessions or even two consecutive identical sessions, typical in split-routine programs [9], or as within-session, for example, the order of exercise that seems to enhance the strength but not the hypertrophic gains [10]. Moreover, between-exercise variables may still be listed, as for example the type of load (e.g. cables, elastic bands, barbells/dumbbells, isoinertial devices) that appear to lead to different short-term or long-term strength and structural adaptations [11–15].

Furthermore, when reporting resistance training protocols, many within-exercise variables should be considered, i.e. variables that refer to any given exercise that may affect the muscle strength and structural adaptations. Typically, the load lifted, absolute or relative to the 1-RM, and the number of sets x repetitions (i.e. the total number of repetitions) sometimes referred to as total volume [16] are usually reported. However, the number of repetitions may be a valid indicator of the total volume of a given exercise, provided that other variables are fixed. The displacement of the load appears to be another variable that should be accounted for: as an example, the squat exercise can have a multitude of ranges of movement, with both acute [17] and chronic [18] differences. With the intent to include the load–displacement into the calculation of total volume, an accelerometer was suggested to match the displacement with force [19]. However, while the availability of an encoder might be a limitation in practice, still equal load displacements can be obtained by different joint angular movement: for example, deep or parallel back-squatting may enhance the hip or knee forces, and consequently muscle activation, when the bar is placed high or low [20]. Additionally, even though a given total displacement is granted (e.g. 90° knee angle motion), such movement could be performed as a partial motion corresponding to long or short muscle length, with possible repercussions on the muscle structure [21]. Therefore, the question is not easily solved. Furthermore, the time under tension, i.e. the sum of the tempos for the concentric, isometric, and eccentric phase [22], performing each set to failure or not even matching for the total number of repetitions [23, 24], the inter-set rest interval [25] or an internal or external focus (i.e. the muscle-mind connection) [26, 27] may have an impact on the neuromuscular and mechanical stimuli derived from the resistance training practice.

Nevertheless, authors have previously failed to report such a detailed description of the exercises performed within the resistance training protocols. Since no one is exempt from incompleteness, in prior studies I have co-authored about the effects of different bench press protocols, we indicated the relative load, the total number of repetitions, the tempo for only the concentric and eccentric phase and the inter-set rest [28]. Moreover, we provided a similar description for comparing the effects of different unilateral leg-extension protocols on the trained [5] or untrained limb [29]. On other occasions, we provided more details to compare the short- [30] or long-term effects of different eccentric-based protocols [31], and we reported the load, the number of repetitions, the range of movement and the duration of the dynamic phases. However, I acknowledge that such descriptions of the resistance exercise are still incomplete. Therefore, the present conceptual review intends to summarize the variables that should be considered when reporting each exercise within resistance training protocols. Incidentally, some of these variables may be also useful to quantify the volume of a given resistance exercise, with the purpose to have a clearer idea of the progression of the stimuli derived from the exercises performed in each training session. With this intent, the importance of each variable will be explored to provide practical indications to both sports scientists and practitioners and describe clearly the technique of a given exercise. Lastly, a checklist will be designed that may serve as methodological guidance for scientific use and training prescription.

## Within-Exercise Variables Affecting Resistance Exercise Volume

### Load

The load is the external resistance the muscle–tendon complex undergoes and represents the acute external mechanical stimulus received by the muscle structures [4]. Typically, the load in resistance training is indicated as the weight lifted (kg) or expressed as %1-RM, i.e. the percentage of the maximum weight lifted once. Among the possible classifications that characterize the amount of load, “high” load is usually considered as > 60%1-RM while a “low” load is $\le$ 60%1-RM [32, 33], even though other classifications may also include “moderate” loads between 60%1-RM and 80%1-RM [34]. Previous studies reported similar increases in strength using high (80%1-RM) versus light load (30%1-RM) in different exercises in previously untrained women performed to failure [35], as well as increases in strength and hypertrophy were observed in recreationally active men after different resistance training protocols performed to failure with 20%, 40%, 60% or 80%1-RM [36]. However, the authors noticed greater hypertrophic effects after the 80% versus 20%1-RM protocol [36]. Other authors found similar hypertrophic but greater strength gains induced by high versus low load in resistance-trained men [37]. More recently, some systematic reviews with meta-analysis have compared the effects between high and low load on different parameters. A first meta-analysis has summarized the effects of high (> 60%1-RM) versus low loads ($\le$ 60%1-RM) training performed to failure by healthy subjects for a minimum duration of six weeks on muscle strength and hypertrophy [32]. The authors showed that strength gains in dynamic 1-RM were greater when high loads were used (effect size: 0.58, 95% CI 0.28 to 0.89), albeit similar gains in isometric strength (effect size: 0.16, 95% CI − 0.10 to 0.41) and hypertrophic effects (effect size: 0.03, 95% CI − 0.08 to 0.14) were retrieved [32]. However, the same authors highlighted that the results were mostly based on inexperienced subjects, since only three out of the 14 studies included enrolled trained people [32]. Another meta-analysis has reported that the hypertrophic effects of high versus low loads are similar in type-I (effect size: 0.28, 95% CI − 0.27 to 0.82) and type-II fibres (effect size: 0.30, 95% CI − 0.05 to 0.66) [33], even though it was previously suggested that the lack of difference may be mediated by other factors such as the training to failure, since in protocols including training not to failure high loads appear more effective to stimulate the increase in the size of all fibre types [34]. Moreover, while similar increases in strength and hypertrophy were observed in elderly people (mean age 70 years, range 64–83) trained for 12 weeks using high (80%1-RM) or low load (40%1-RM), the former increased the pennation angle, while the latter was effective to increment the initial rate of force [38]. Taken together, high and low loads may be both effective for resistance training, provided that other factors (e.g. training to failure) are accounted for. However, high loads appear more suitable to maximize the strength gains. Therefore, load is a primary factor to be reported and should be included in the calculation of the total volume of an exercise.

### Total Number of Repetitions

The number of repetitions can be considered as the number of times the force produced by the muscle–tendon complex manages the external load. As for the load, the total number of repetitions is very likely indicated in each study investigating the effects of resistance training. Although it might appear that more repetitions may induce more adaptations, the studies were not always in line with this dose–response assumption. For example, when increasing the number of repetitions by adjusting the number of sets performed for each exercise, similar strength gains were observed between 1 ×, 3 × or 5 × 8–12 repetitions [16]. However, the authors found a graded dose–response relationship regarding the hypertrophic adaptations [16]. Interestingly, when searching for the minimum total number of repetitions required to increase strength in resistance-trained men, a total of 6–12 repetitions per exercise performed twice a week appeared a sufficient stimulus [39]. Notwithstanding, the hypertrophic stimulus resulted in an overall dose–response relationship irrespective of sex, age, and body region [40]. To summarize, the total number of repetitions must be indicated, primarily because the dose–response principle may be valid for the muscle hypertrophic but not for the strength adaptations. Incidentally, the total number of repetitions should be considered as part of the volume of an exercise and should not be considered as volume itself.

### Repetitions to Failure/Not to Failure

The total number of repetitions for a given exercise can be distributed in multiple ways across the sets, irrespective of the load. However, the number of repetitions within a set is strictly connected with the load since low loads allow choosing a wide range of repetitions performed in a single set, while high loads do not. In this sense, the maximum number of repetitions per set (i.e. performing a single set to failure) decreases with the increment in the load [41]. The rationale for training to failure, more specifically until the load can be lifted concentrically, is to enhance the muscle recruitment [41, 42], albeit this was not confirmed when muscle activation was investigated comparing heavy loads versus repetitions to failure [43]. Nevertheless, performing repetitions to failure seems to promote greater metabolic impact at cellular level [44]. In the practice, repetitions to failure are often used to lead to greater total time under tension, possibly increasing the total volume [23]. On the other hand, repetitions to failure may need more time to recover and not be comfortable in less experienced people [45]. Recently, two different meta-analyses compared the effects of repetitions to failure versus not to failure on strength and hypertrophy [23, 24]. When the total volume was not matched (albeit not declared how), one meta-analysis reported greater overall increases in strength (effect size: 0.34, 95% CI 0.00 to 0.67) and power (effect size: 0.61, 95% CI 0.08 to 1.15) when training was performed not to failure and greater hypertrophic response when training was performed to failure (effect size: 0.82, 95% CI 0.09 to 1.56) [23]. Notwithstanding, the same authors observed that all these differences disappeared when the volume was matched, making the two training methods equivalent [23]. The other meta-analysis found no difference in strength and hypertrophy gains when training to failure versus not to failure, even though greater strength gains were reported after training not to failure (effect size: 0.32, 95% CI 0.07 to 0.57) when the volume was not equated (albeit not declared how), while the training experience and the body region did not influence the strength gains [24]. Notably, the authors reported a small but significant advantage in the hypertrophic adaptations in resistance trained individuals when training to failure (effect size: 0.15, 95% CI 0.03 to 0.26) [24]. Possible methodological differences in the studies' inclusion criteria may explain the apparently not univocal results, such as the inclusion of both young and older adults in the former [23] but not the latter meta-analysis [24] and the randomization of the participants required to be included in the latter [24], so that these factors should be accounted for when drawing conclusions. Nonetheless, although caution is needed, repetitions to failure might enhance the hypertrophic process, while not to failure might increase the strength gains. However, as both meta-analyses have highlighted, equating the training volume may lead to equivalent results, making necessary a clear understanding of what “volume” is made of. Lastly, performing repetitions to failure appeares as a primary stimulus when light loads are used, as discussed above. On these bases, it seems necessary to report whether a given total number of repetitions has been obtained performing repetitions to failure or not. Remarkably, performing repetitions to failure or not to failure does not affect the volume of an exercise per se, but is necessary to fix the context in which a given number of repetitions is performed.

### Displacement/Range of Movement

The displacement of the external load is the result of the combined movements of all joints involved in each exercise that also depict the trajectory of the external load. While the displacement per se includes a start and an end, during the traditional resistance training execution where both concentric and eccentric phases are performed the external load continues from the start to the end of the movement and vice versa, so that the displacement includes both phases. In single-joint exercises, the displacement of the external load overall coincides with the joint angular range of movement (e.g. the displacement of a dumbbell during a biceps curl mostly derives from the range of movement at the elbow joint), while in multi-joint exercises, the displacement of the external load is due to the movement of different joints that regulate partly or totally the trajectory of the external load (e.g. squatting depends at the very least on the combination of the flexion/extension of the hips, knees and ankles). Practitioners usually prescribe resistance exercises with a full or partial range of movement; therefore, should all other variables be equated, a full range of movements implies greater displacement of the external load, thus more work and volume [46]. On the other hand, most of the exercises include a so-called “sticking point” (or sticking region), i.e. a point or portion of the movement where there is a biomechanical disadvantage and momentary failure will occur [47]. Partial movements may overcome the sticking point and let higher loads be used, albeit full ranges are expected to be associated with greater time under tension [46]. Acutely, when equated for the relative load, some authors found greater activation of gluteus maximus, biceps femoris, and soleus during partial (0–90° knee flexion) versus full back-squat (0–140° knee flexion), while no difference in the activation of quadriceps and erector spinae was observed [48]. Other authors did not observe any difference in the activation of gluteal, thigh, and back muscles comparing parallel versus full back-squat performed with 80%1-RM of the relative load, albeit this may depend on the less pronounced difference in terms of displacement between the two exercises [17]. Nevertheless, when partial (0–90° knee flexion) versus full back-squat (0–140° knee flexion) was performed for 10 weeks, differences emerged [18]. In the first instance, the strength gains in full 1-RM were greater in the full versus partial group, while the gains in partial 1-RM were similar [18]. Furthermore, the volume of gluteus maximus and adductor muscles increased more when full versus partial back-squat was performed, with no difference in hypertrophic gains of the quadriceps [18]. Notwithstanding the non-uniform regional hypertrophic adaptations induced by partial versus full movements reported in a recent systematic review [46], the authors estimated similar overall hypertrophic stimulus. However, this is mediated by other variables such as muscle activation and the different relative load. Therefore, because of the non-uniform stimuli induced by partial versus full movements, the displacement of the external load or the range of movement of a meaningful joint (e.g. knee for squat) should be reported. It should be remembered that the displacement of the external load should be considered as part of the exercise volume calculation, also possibly determined as the range of movement of the main joint involved in the exercise.

### Same Partial Displacement, Different Muscle Length

Let us suppose we have reported that partial range of motion, e.g. 90° elbow flexion/extension in a biceps curl will be performed (please note that the present section does not apply to exercises performed with full ranges). However, even though the volume per se is so far equated, the region within the range of movement where the exercise is performed can affect the acute and chronic results. Indeed, every exercise contains theoretically a sticking point (or region) where the load is more difficult to be lifted [47]. Although the nature of the sticking point has been described as multifaceted, biomechanical disadvantages and the muscle-length relationship have been advocated to explain it [47]. Incidentally, the sticking point of the single-joint exercises is mostly dependent on the characteristics of the single muscle–tendon group involved in the movement, while in multi-joint exercises it depends on the overall combination of the characteristics of all muscle–tendon groups simultaneously acting during the exercise. In the example of a biceps curl, one can perform a range of movement equal to 90° flexion/extension as 0–90° (0° = full extension) or 45°–135°. Considerations for more complex yet widely used exercises such as bench press and squat have been made [49]. As the sticking point affects the momentary failure [47], reporting whether or not it falls back into both ranges or one of them is relevant, given that less or more numerous repetitions to exhaustion may consequently be performed. In addition to the performance, the sticking point may also be connected to an increased risk of injury, as somewhat remarked [49]. Further considerations can be made about the muscle length at which partial ranges are performed. For example, short versus long muscle length was associated with less fatigability because of lower peripheral fatigue [50], and the ability to perform maximum contractions at long muscle length can be trained [51]. Additionally, inconsistency in muscle damage was reported after eccentric exercise at various muscle lengths, with greater damage observed at short [52] or long muscle length [53]. Lastly, hamstrings training performed at long or short muscle length resulted in an enhancement of the fascicle elongation, albeit similar strength and hypertrophic gains [21]. Taken together, when partial ranges of movements are performed, the authors should report whether these correspond to movements executed at long or short agonist muscle length.

### Time Under Tension

When performing an exercise listing all parameters above, the time under tension for each repetition has not been still considered. Provided that each repetition includes four phases (the first isometric, the concentric, the second isometric and the eccentric phase, with the possibility to switch the two dynamic phases, e.g. in the squat), the time under tension is the sum of the tempos necessary to perform each phase [54]. Alternatively, as specifically concerns the concentric and eccentric phase, the tempo depends on the velocity at which the external load is displaced [55]. Increasing the time under tension can be considered a way to augment the volume [19], and it is a common belief that increasing the time under tension might be a strategy to enhance the hypertrophic stimulus [56].  However, fast movements need more neural control and depend on the capacity to maximally recruit the highest number of motor units [57]. Moreover, fast versus slow eccentric phases resulted in greater muscle damage when total time under tension is equated, so that more recovery is needed between sessions [58, 59]. Additionally, since muscle damage is considered one of the factors that initiate the hypertrophic process [56], fast movements may be more effective, contrary to what is believed in the practice. Another study compared the chronic effects of 1–0–1 s versus 1–0–3 s tempo for the concentric, isometric, and eccentric phases, respectively [55]. After the 8-week intervention, strength and the proximal muscle size increased similarly in both groups, even though the hypertrophic response at the distal site was more pronounced after the fast protocol [55]. The authors also observed lower perceived effort during fast versus slow training [55]. This may have many practical repercussions since some people may not like the feeling of too much effort during training, reducing their adherence. Concerning the gains in dynamic strength, a recent meta-analysis observed overall similar results between fast versus slow movements (effect size: 0.07, 95% CI − 0.13 to 0.27) [60]. However, the authors noticed that fast movements were possibly more beneficial (effect size: 0.31, 95% CI − 0.01 to 0.63) when moderate loads (60–79%1-RM) were used, while there were no difference between fast and slow training with low (< 60%1-RM, effect size: − 0.06, 95% CI − 0.45 to 0.32) or high loads (> 80%1-RM, effect size: − 0.08, 95% CI − 0.41 to 0.25) [60]. Another systematic review without a meta-analysis summarized the effects of fast versus slow movements on muscle hypertrophy, considering the exercises involving upper or lower body muscles with a special reference to biceps brachii and quadriceps, respectively [61]. Intriguingly, fast movements appeared to enhance the increment in the size of the biceps brachii, while slow movements were more advantageous for quadriceps [61]. As the authors discussed, this may depend on the difference in fibre-type prevalence between the two muscle groups, with more type-II fibres in biceps brachii than quadriceps, leading to the idea that specific movement velocity strategies may be used depending on the muscle morphology [61]. Notably, the studies included in this previous meta-analysis manipulated both the concentric and the eccentric phase so that the results were not related to the intrinsic characteristics of the shortening versus lengthening contraction [61]. This further highlights that the time under tension is a necessary variable to be listed when reporting resistance training protocols and should be considered in the exercise volume determination. Intriguingly, the analysis of the relevant literature questions the common belief that slow movements favour hypertrophy.

Although most attention is on the duration of the two dynamic phases, it is also worthwhile examining the two isometric actions within each repetition. Firstly, one isometric phase usually occurs in a “comfortable” position, where the external resistance is minimal because of biomechanical advantages (e.g. extended forearm in biceps curl); the second isometric phase occurs at the end of the first dynamic phase, irrespective of whether partial or full movement has been performed, where the external resistance for a given load is much greater compared to the first one. Some sports, such as powerlifting, require an identifiable second isometric phase to perform a valid lift, so many practitioners tend to insert it within each repetition. In the first instance, varying the position at which the second isometric phase is performed is associated with the length of the muscles involved in the movement. Although no study has directly investigated the effects of the isometric phase at different muscle lengths, a recent review examined this factor when isometric-only training is performed, providing useful indications [62]. For example, greater hypertrophic stimulus derived from isometric training matched for duration performed at long versus short muscle length [62]. Moreover, more pronounced changes in muscle architecture were induced at long versus short muscle length, as well as greater improvements in tendon function [62]. Therefore, the position at which the isometric phase occurs may influence the muscle structure and should be reported. Another aspect is the duration of each isometric phase. Basically, longer durations lead to greater accumulation of metabolites, possibly increasing the hypertrophic stimulus [63]. Interestingly, some practitioners tend to perform the isometric phase to exhaustion, resulting in a so-called eccentric quasi-isometric exercise [63]. Though this method was proposed decades ago, its scientific soundness has been examined only recently [63], and its effects have been poorly investigated. It is known that eccentric quasi-isometric training can maximize the mechanical tension at short and moderate muscle length [64], and that muscle damage is less compared to eccentric-only training [65]. However, long-term investigations are still lacking and are warranted to further elucidate the topic. To summarize, the position of the two isometric phases should be reported, since possible different effects especially on muscle structure may derive.

### Concentric Versus Eccentric Versus Concentric/Eccentric Phases

Traditional resistance training is routinely performed with the execution of both the concentric and the eccentric phase. However, an interest in both the acute and chronic effects of the concentric- or eccentric-based training has been described. Performing the eccentric phase only allows greater loads to be lifted [28] because of its unique mechanical [66] and neuromuscular characteristics [67] compared to the concentric phase. Short-term studies reported muscle damage markers mainly induced by a single eccentric-based session [68], albeit such damage is essential to provide muscles with a protective effect from subsequent eccentric exercise, i.e. the repeated-bout effect [69]. Chronically, although not all the included studies matched the training protocols for total volume, different meta-analyses reported possible advantages in strength [70, 71] and hypertrophic gains [70, 72] for the eccentric- versus concentric-based training. Interestingly, eccentric- or concentric-based training is associated with different typical adaptations in muscle architecture, the former inducing a more pronounced fascicle elongation, while the latter results in increments in pennation angle [73]. However, while the eccentric- versus concentric-based training has been gaining much attention, the inclusion of traditional concentric-eccentric protocols into the comparison is scarce. In a study where the training volume was matched for the combination of number of total repetitions, load, displacement, and total time under tension, the authors reported that concentric-based, eccentric-based, and traditional concentric-eccentric resistance training performed by trained men led to similar gains in bench press 1-RM, albeit the eccentric-based was the only protocol to induce hypertrophic effects and improve muscle-endurance, and to maintain all adaptations after a detraining period [28]. Using a more comprehensive design, another study reported that iso-load concentric-based, eccentric-based, and traditional concentric-eccentric protocol led to increases in concentric, isometric, and eccentric strength, albeit the eccentric strength gains were lower after the concentric-based training [5]. Moreover, only the inclusion of the eccentric phase stimulated the fascicle elongation and all protocols induced a widening in pennation angle, while the hypertrophic stimulus was greater when the eccentric phase was included [5]. Lastly, the retention of the results after a detraining period was more marked when performing the eccentric phase [5]. For all the aforementioned reasons, it should be reported whether the exercise includes the execution of both the concentric and the eccentric phase or just one of them. Incidentally, performing one or two phases has repercussions on the total displacement of the external load, and therefore should be considered in the calculation of the exercise volume.

### Internal Versus External Attentional Focus

The so-called “muscle-mind connection” is the capacity to over-activate some muscles during an exercise or a task by “thinking” of it during the movement [26]. When performing the bench press, we could decide to have an attentional focus on a given muscle (internal focus) or on the whole movement or task (external focus) [27]. For example, in the case of bench press, we could have an internal focus on pectoralis major or triceps brachii, or an external focus on the lifting. Such a training strategy is quite well known in the practice, with the idea to elicit the activation of one muscle group during a given exercise, thus increasing its work and possibly the hypertrophic stimulus. Therefore, the first step is to understand whether or not the internal focus increases muscle activation compared to an external focus with the same relative load. The literature is in general favourable to this, as reviewed over the years [26, 27], even though the experience of the subjects [74], the muscles involved [75], the movement velocity [76], or the load [77, 78] may affect the results. The studies report greater muscle activation with an internal focus on several muscles during different exercises, such as pectoralis major during bench press [77], biceps brachii during biceps curl [79], quadriceps during leg extension [80], or posterior thigh muscles during the squat [78]. Among the factors that may influence the ability to over-recruit the muscles through an internal focus, two merit an in-depth analysis for the implications in practice. First, more experienced may be more able than less experienced people to increase muscle activation [74]. Second, very high external loads require yet higher muscle activation, so it may be difficult to further recruit muscles compared to light loads, where more marked increments in muscle activation were observed [77]. However, experienced subjects were able to increase muscle activation of the triceps brachii during bench press [75] or gluteus maximus and biceps femoris during the squat [78] with both 50% and 80%1-RM. Nevertheless, the coin has two sides. Indeed, while increasing the muscle activation, an internal focus showed equal or detrimental effects on muscular endurance [81] and strength [79, 80], as also recently summarized in a meta-analysis [82]. In other words, muscle efficiency decreases [27], albeit this might not be a problem when searching for an enhancement of the hypertrophic stimulus, the main purpose of practitioners that use the attentional internal focus. To check for the truthfulness of this assumption deriving from the practice, only one study has compared to date the effects of an internal versus external focus systematically used during an 8-week resistance training protocol [83]. Two muscle/movement couples were examined: biceps brachii for biceps curl and quadriceps for knee extension, and both strength and hypertrophic changes were recorded [83]. While strength gains were similar between the internal and external focus in both exercises, the hypertrophic gains in biceps brachii were more pronounced with the internal focus, while no between-focus difference was found in quadriceps [83]. On these bases, considering the possible acute and chronic differences between the internal versus external focus, the attentional strategy should be always reported. Of note, this may not affect the volume of an exercise considered as the total external resistance but may be considered a way to increase the internal load, i.e. the stimulus provided to a given muscle.

### Inter-Set Rest

The inter-set rest is the time between two sets of the same exercise or between two exercises. Here, the former will be considered since the within-exercise context is examined. In the practice, short is claimed to favour the hypertrophic response via an increased metabolic stimulus, while longer inter-set rest duration should generally favour the gains in the force-generating capacity [25]. However, before examining the effects of different durations of the inter-set rest, it should be first specified whether the rest is passive or alternative strategies are performed. A recent systematic review has examined the effects of agonist/antagonist stretching, cooling, aerobic exercise, vibration, and individualized heart-rate intervals on acute performance [84]. The results were heterogeneous, and it should be noted that acute but not chronic effects were collected [84]. However, distinguishing between passive or active rest is the first step. Chronically, trained individuals may benefit from rest longer than 2 min even though uncertain results may derive above 5 min, while untrained people appear to have similar strength gains irrespective of the inter-set rest duration [25]. Furthermore, multi- versus single-joint exercises may need different rest durations, so the authors concluded that the inter-set rest time should be tailored [25]. A meta-analysis of the hypertrophic response is also available [85]. Overall, the studies indicate that inter-set rest durations longer than 1 min are more advantageous for increasing muscle size, even though the authors also mentioned the hypothesis that the greater metabolite accumulation with shorter rest may be used to alternate the stimulus and maximize the hypertrophic process [85]. Therefore, the inter-set rest duration should always be indicated along with the type of rest. Notably, the inter-set rest does not influence the exercise volume but may have both acute and chronic repercussions.

## Conclusion

**Fig. 1:**
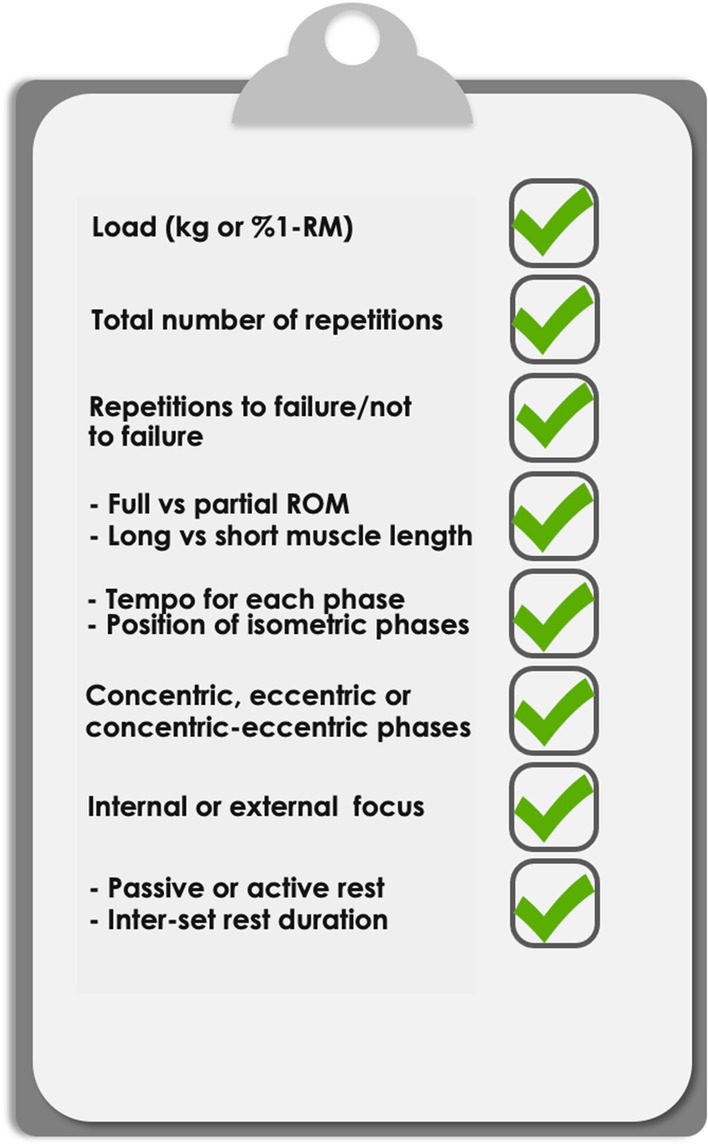
A checklist of the variables to be reported for each exercise within the resistance training protocols

Appropriate reporting of the exercise variables in resistance training protocol includes a number of within-exercise variables, as examined here and shown in Fig. 1. The present conceptual review summarized all variables that should be listed, explaining why each variable is relevant and important.

**Fig. 2:**
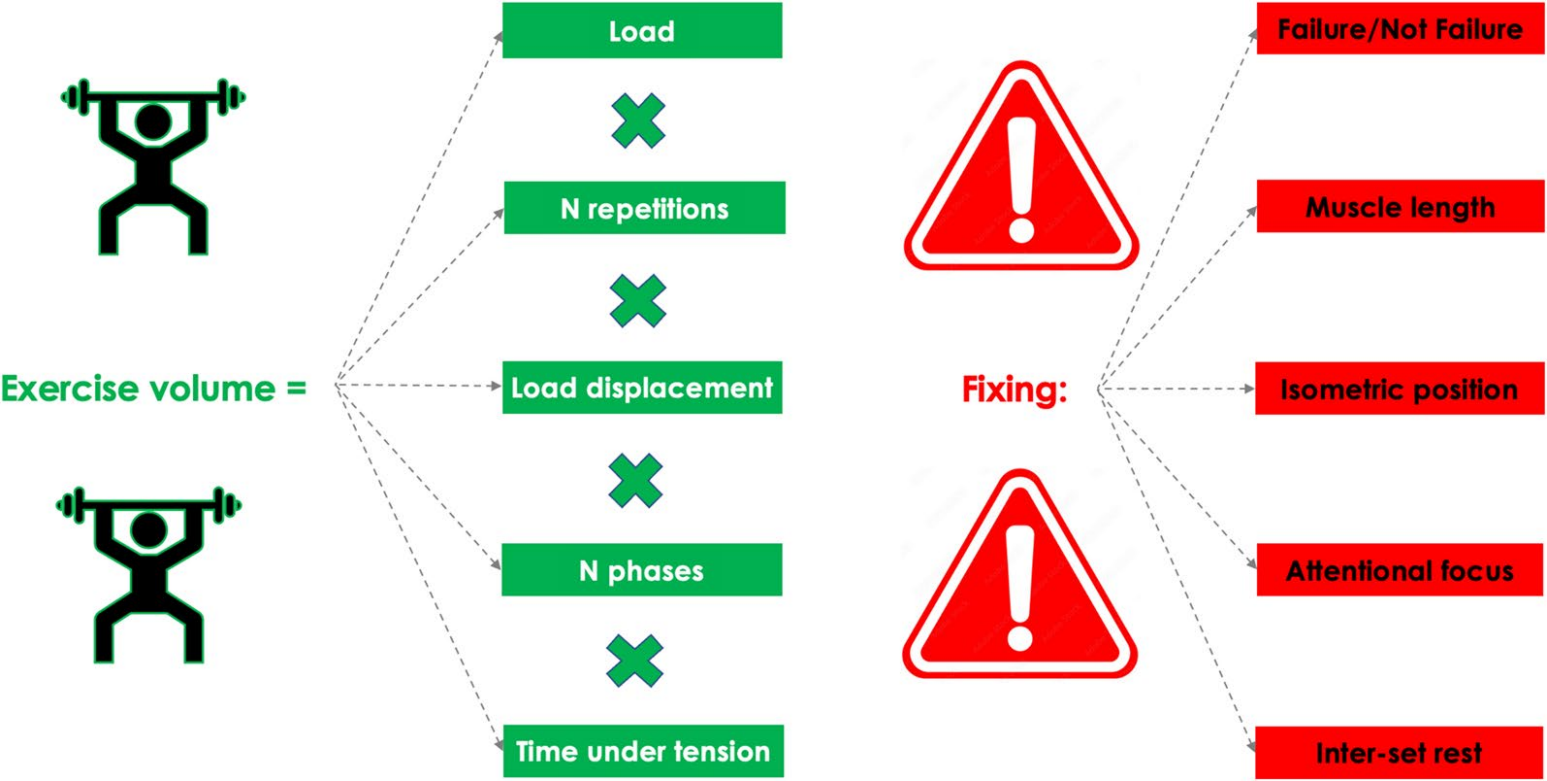
A more comprehensive approach to the calculation of the volume of a single exercise. The variables in green determine the volume, provided that the variables in red are fixed

Moreover, quantifying the volume of each resistance exercise requires the specification of within-exercise variables further than the load and the number of repetitions, while the number of repetitions should not be considered as a synthetic way to indicate the volume of an exercise. Remarkably, listing the aforementioned variables also provides a detailed description of the technique used for each exercise. Using the present and more comprehensive approach, sports scientists and practitioners may be able to quantify the volume of each exercise properly. By doing so, modifying one or more of the variables will allow to increase, equalize or decrease the stimuli induced by a single exercise. The variables directly affecting the exercise training volume and the variables that should be fixed for an appropriate comparison are shown in Fig. 2.

It is important to note that these variables refer to a within-exercise context and are not indicative of all further variables that can be controlled in each session or micro-cycle.

## Data Availability

Not applicable.
